# Production of a Thermostable and Alkaline Chitinase by *Bacillus thuringiensis* subsp. *kurstaki* Strain HBK-51

**DOI:** 10.1155/2012/135498

**Published:** 2012-12-13

**Authors:** Secil Berna Kuzu, Hatice Korkmaz Güvenmez, Aziz Akin Denizci

**Affiliations:** ^1^Biotechnology & Molecular Biology Division, Department of Biology, Cukurova University, 01330 Adana, Turkey; ^2^Research Institute for Genetic Engineering and Biotechnology, The Scientific and Technical Research Council of Turkey (TUBITAK), Marmara Research Center Campus, Gebze-Kocaeli 41470, Turkey

## Abstract

This paper reports the isolation and identification of chitinase-producing *Bacillus* from chitin-containing wastes, production of a thermostable and alkaline chitinasese, and enzyme characterization. *Bacillus thuringiensis* subsp. *kurstaki* HBK-51 was isolated from soil and was identified. Chitinase was obtained from supernatant of *B. thuringiensis* HBK-51 strain and showed its optimum activity at 110°C and at pH 9.0. Following 3 hours of incubation period, the enzyme showed a high level of activity at 110°C (96% remaining activity) and between pH 9.0 and 12.0 (98% remaining activity). Considering these characteristics, the enzyme was described as hyperthermophile-thermostable and highly alkaline. Two bands of the enzyme weighing 50 and 125 kDa were obtained following 12% SDS-PAGE analyses. Among the metal ions and chemicals used, Ni^2+^ (32%), K^+^ (44%), and Cu^2+^ (56%) increased the enzyme activity while EDTA (7%), SDS (7%), Hg^2+^ (11%), and ethyl-acetimidate (20%) decreased the activity of the enzyme. *Bacillus thuringiensis* subsp. *kurstaki* HBK-51 is an important strain which can be used in several biotechnological applications as a chitinase producer.

## 1. Introduction

Chitin, a linear *β*-(1,4)-linked *N*-acetylglucosamine (GlcNAc) polysaccharide, is the main structure component of the fungal cell wall and the exoskeletons of invertebrates, such as insects and crustaceans. Chitinase plays a variety of important roles in these organisms ranging from nutrition to defense and control of ecdysis in insects. The importance of chitinases in many biological processes makes their inhibitors important targets for potential antifungal and insecticidal agents as well as antimalarial agents [1]. 

It is one of the most abundant naturally occurring polysaccharides and has attracted tremendous attention in the fields of agriculture, pharmacology, and biotechnology. Chitin is the second most abundant component of biomass on earth [2]. This linear polymer can be hydrolyzed by bases, acids or enzymes, such as lysozyme, some glucanases, and chitinase. Chitinases (EC 3.2.1.14), essential enzymes catalyzing the conversion of chitin to its monomeric or oligomeric components (low-molecular-weight products), have been found in a wide range of organisms, including bacteria, plants, fungi, insects, and crustaceans. Because chitin is not found in vertebrates, it has been suggested that inhibition of chitinases may be used for the treatment of fungal infections and human parasitosis [3]. Biological control, using microorganisms to repress plant disease, offers an alternative environmentally friendly strategy for controlling agricultural phytopathogens [4]. The production of inexpensive chitinolytic enzymes is an important element in the utilization of shellfish wastes that not only solves environmental problems but also promotes the economic value of marine products, so chitinases have been studied and purified from many microorganisms, and their enzymatic properties have been investigated [5]. Global annual recovery of chitin from the processing of marine crustacean waste is estimated to be around 37.300 metric tons [6]. 

## 2. Materials and Methods

### 2.1. Chemicals

Chitin-Azure was (Sigma-Aldrich), PMFS, Protein Marker (BSA), CBB R-250, DMSO and the others were purchased from Merck (Germany).

#### 2.1.1. Isolation of Bacteria

Crabs, campus soil, and compost were mixed and held to approximately 30 days outside (exposure sun, rain or etc.). After this period, sterile distilled water was added to material and homogenized. 10 mL supernatant wae transferred to glass tube and incubated at 80°C for 10 min to eliminate vegetative forms of bacteria. 1 mL sample inoculated to 10 mL LB and incubated at 37°C for 6 hours and then sample was diluted 10^−6^ − 10^−7^ fold with freshly prepared LB, then streaked on N1 agar plate. After incubation at 37°C overnight, single colonies were selected (550 colonies in total) and stock cultures prepared [7].

### 2.2. Preparation of Colloidal Chitin

Preparation of colloidal chitin was performed from commercial chitin (C9752, Sigma-Aldrich Co, USA) according to Wen et al. [3] with minimal modifications. Fivegrams of chitin powder were added into 60 mL of concentrated HCL and left in refrigerator (at 4°C) overnight with vigorous stirring. The mixture was added to 2 litres of ice-cold Et-OH (95%) with rapid stirring and kept overnight at room temperature (at 22°C). The precipitate was collected by centrifugation at 6,000 ×g (4°C) for 25 min. The precipitate was washed with sterile distilled water until the colloidal chitin became neutral (pH 7.0) and then stored and at 4°C for further applications. 

### 2.3. Screening Chitinase Producer Strains

All strains (total 550) were selected from soil including chitin wastes, tested one by one for chitinase activity on the CHDA (chitinase-detection agar). Chitinase producer strains were determined after 3–5 days at 37°C by visualizing the clear zone formed surrounding of the colonies on the CHDA plate. CHDA agar, used first detection of chitinase positive strains and preparation of stock culture, by adding 10% g of colloidal chitin and 20 g of agar in M9 medium containing (g/L); 0.65 NaHPO_4_, 1.5 KH_2_PO_4_, 0.5 NH_4_Cl, 0.12 MgSO_4_, 0.005 CaCl_2_, 0.25 NaCl, pH = 6.5 [3, 8].

### 2.4. Enzyme Production

HBK-51 strain (*Bacillus thuringiensis* subsp. *kurstaki*) was used as chitinase producer. 1 mL of HBK-51 strain fresh culture was inoculated into 10 mL of LB medium (containing 1% colloidal chitin as the sole source of C and N) and incubated overnight at 37°C with shaking (150 rev min⁡^−1^). The culture was transferred into a 2 litres glass bottle containing 500 mL M9-Medium which was supplemented with 1% colloidal chitin and incubated at 37°C on a rotary shaker at 150 rev min⁡^−1^ for 3 days [3]. The culture was centrifuged for 20 min at 9000 ×g, at 4°C and supernatant was separated from cell debris. Then supernatant was filtered (with filter paper) and precipitated with Et-OH (ethyl-alcohol) [9]. All experiments were done with this enzyme preparation.

### 2.5. Purification of the Chitinase

Proteins in the culturesupernatant fluid were precipitated with Et-OH (ethyl-alcohol) at −33°C overnight. Et-OH (ethyl-alcohol) was added 70% of the original volume of supernatant. The precipitate was recovered by centrifugation (12,000 ×g (4°C) for 30 min.), dissolved in a small amount of 0.1 M Na-phosphate buffer (pH 7.0), and stored at 4°C [10, 11].

### 2.6. Measurement of Enzyme Activity

Chitinase activity was measured with chitin-azure as a substrate [12]. Enzyme solution (0.5 mL) was added to substrate solution (0.5 mL), which contained 1.0% chitin azure in a sodium acetate buffer (100 mmol/L, pH 7.0), and the mixture was incubated at 50°C for 30 min. After centrifugation (12,000 ×g, 15 min, at 4°C), enzyme activity was measured at 595 nm absorbance using spectrophotometer. One unit of chitinase was the amount of enzyme that produced an increase of 0.01 in the absorbance [12, 13].

### 2.7. pH and Temperature Optima

The optimum pH and temperature were determined by incubation in a buffer at different pH values (3.0–10.0) and temperatures (30–120°C) under standard assay conditions using chitin-azure as the substrate. The buffers used were 50 mM sodium acetate buffer (pH: 3.0–6.0), 50 mM Tris-HCl buffer (pH: 7.0–9.0), and 50 mM Glycine-NaOH buffer (pH: 10.0–12.0). The optimum temperature for enzyme activity was measured under standard assay in the range of 30–120°C with intervals of 10°C [14]. 

### 2.8. Thermal Stability

The effect of the temperature on the stability of chitinolytic enzyme was determined by exposure of the enzyme solution in 50 mM Tris-HCl buffer, pH: 9.0 (optimum pH) to different temperatures (30–110°C) for 3 h. The residual activity was then assayed under standard conditions using chitin-azure as the substrate [14, 15].

### 2.9. pH Stability

For pH stability assay, the enzyme solution was preincubated for 3 h at room temperature in the buffers of various pH values (pH: 5.0–12.0) and then the residual activity was determined under standard conditions [14, 15].

### 2.10. Plasmid Curing

All bacteria which produced chitinase were tested with EtBr for plasmid elimination according to Hardy [16]. 

### 2.11. Identification of *Bacillus* sp

Criteria used for classification and identification of *Bacillus* strain HBK-51 were based on morphological [7], physiological and biochemical tests (Table 1) [17], fatty acid analyses (FAME) (Sherlock-MIDI Automated Microbial Identification System version 4.0, MIDI inc., Newark, DE), and 16S RNA/DNA sequence analyses [14, 18, 19].

### 2.12. 16S rRNA Gene Sequence Comparison

The 16S rRNA gene was amplified by polymerase chain reaction (PCR) with forward primers:

 27F 5′-AGAGTTTGATCMTGGCTCAG-3′ (8–27 position),
 530F 5′-GTGCCAGAGCMGCCGCGGTAA-3′ (515–533 position)

and reverse primers:

 1525R 5′-AAGGAGGTGWTCCRCC-3′ (1541–1525 position)
 1100R 5′-AGGGTTGCGCTCGTTG-3′ (1115–1100 position).

The amplified PCR product was sequenced by the Beckman Coulter-CQ 8800 model sequence analyzer with their methods. The 16S rDNA sequence was aligned with other 16S rDNA bacterial sequence obtained from GenBank by basic local alignment search tool (BLAST) program [20].

### 2.13. The Influence of Chemicals (Metal Ions, Chelators, and Detergents)

The chemicals were used in two different concentrations (Table 2). These chemicals' effect on chitinolytic activity was determined by incubating 0.5 mL enzyme with 1–5 mM EDTA, PMSF, 1,10-Phenanthroline, ethyl-acetimidate, Phenol Gliaksol, N-Ethylmaleimide, Urea, Na_2_SO_3_, NaCl, CaCl_2_, ZnCl_2_, MgCl_2_, BaCl_2_, FeCl_3_, MnCl_2_, CuCl_2_, CoCl_2_, NiCl_2_, KCl, HgCl_2_, and 1–5% SDS and DMSO for 30 min at room temperature after which the residual activity was measured with standard assay [21]. 

### 2.14. SDS-PAGE Analyses

The enzyme molecular weight was determined with sodium dodecyl sulphate polyacrylamide gel electrophoresis (PAGE) [22]. The gel was prepared using 5% stacking gel and 12% of separating gel (Acr : Bis = 29 : 1). After electrophoresis gel was stained with CBB-R 250 then visualized by placing the gel to the Minibus Gel Apparatus, BSA (Sigma) was used as a MW marker.

## 3. Results and Discussion

**Figure 1:**
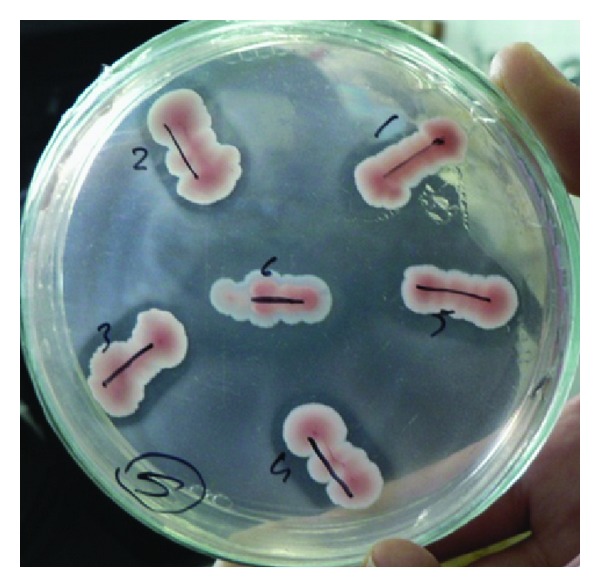
Chitinase activity on CHDA plate.

Chitinase producing bacteria were isolated from chitin wastes on CHDA (chitinase detection agar, Figure 1) and chitinase was produced in the media containing 1% of colloidal chitin [3]. In total, 550 strains were selected from chitin wastes and tested for chitinolytic activity. After screening the strains for their ability to utilize chitin, 12 of them (2.18%) showed chitinolytic activity. 6 strains were showed the highest hydrolysis zone (4–10 mm) on CHDA agar. They were termed HBK-30, HBK-36, HBK-37, HBK-42, HBK-43, and HBK-51. HBK-51 strain was selected as chitinase producer. 

The HBK-51 strain was identified according to morphological and physiological characteristics (are presented in Table 1) [17]. According to Bergey's Manual of Systematic Bacteriology [7], strain HBK-51 was classified as a bacteria belonging to the genus *Bacillus*. Further sequence analysis of the gene encoding 16S rRNA and fatty acid analyses (FAME, data not shown) confirmed the isolate as being *Bacillus* genus and according to BLAST confirmation, it was *Bacillus thuringiensis* subsp. *kurstaki. *Theamplified16S rRNA sequence was submitted to GenBank for possible identification. The result showed 99.00% identity with *Bacillus thuringiensis* subsp. *kurstaki.* The accession number is EU153549. Chitinolytic activity of HBK-51 on CHDA is shown in Figure 1.

According to the plasmid-curing tests, the gene of encoding chitinase of* Bacillus thuringiensis* subsp. *kurstaki *HBK-51 was located on chromosomal. 

### 3.1. pH and Temperature Optima

The pH effect on the chitinase activity was determined using three buffer systems at various pH values. The HBK-51 chitinase was active at broad range of pH (3.0–10.0) but had optimum activity at pH 9.0 (data shown Figure 5), when assayed with chitin azure as a substrate. On the other hand, enzyme was active from 30 to 120°C and exhibited maximum activity at 110°C (Figure 3). The enzyme was stable (after 3 hours incubation period) at 30–120°C and pH 9.0–12.0 and generally, protected original activity approximately 92.4% (Figure 4) and 98% (Figure 6), respectively, at 90°C chitinase was 100% active and at 100–110°C had shown 96% retain activity. Therefore, HBK-51 chitinase was called as thermostable at high temperatures. Siwayaprahm et al. [23] reported that for *Bacillus circulans* No. 4.1. recombinant chitinase, the optima pH and temperature were 7.0 and 45°C, respectively, and it was stable in the pH range of 5.0–9.0 and at temperatures up to 50°C. Lee et al. [24] reported that *Bacillus* sp. DAU101 chitinase had optimum activity pH 7.5 and 60°C, Wen et al. [3] reported chitinase activity of *Bacillus *sp. NCTU2 had in the range of 50–60°C at pH 7.0. Kim et al. [14] reported *Serratia* sp. KCK chitinase had broad range of pH (5.0–10.0) with an optimum value 0f 8.0 and 40°C, respectively. Li et al. [25] reported *Bacillus cereus* strain CH2 had optimum activity at pH 7.1 and temperature at 40°C. According to these results, *Bacillus thuringiensis* subsp. *kurstaki* HBK-51 chitinase is a thermotolerant and alkaline enzyme. Guo et al. [26] reported that *Thermomyces lanuginosus* SY2 chitinase exhibited optimum catalytic activity at pH 4.5, 55°C and enzyme was stable at 50°C and its half-life time at 65°C was 25 min. On the other hand, they reported that the thermostable chitinase had major advantages over industrial catalysis for its high activity at high temperature. Thermostable chitinase could be useful for the chitin industry and biotechnological applications.

### 3.2. Influence of Chemicals and SDS-PAGE Analyses

**Figure 2:**
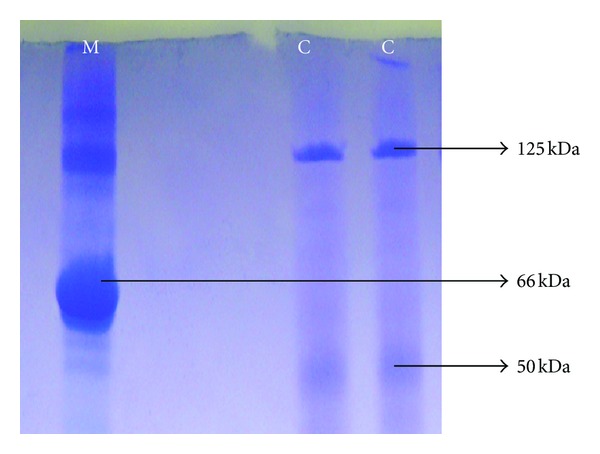
Chitinase molecular weight pattern of *Bacillus thuringiensis* subsp. *kurstaki* strain HBK-51. M: Protein Marker (BSA, 66 kDa), C: Chitinase.

**Figure 3:**
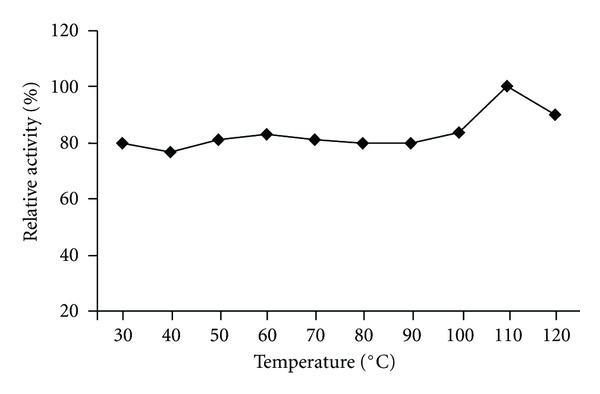
Effect of temperature on the chitinase activity.

**Figure 4:**
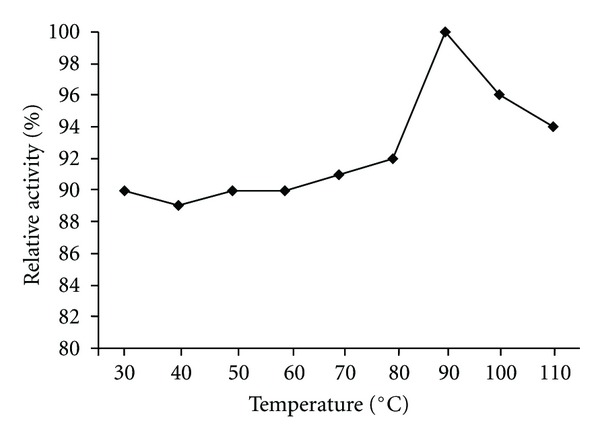
Thermal stability of chitinase.

**Figure 5:**
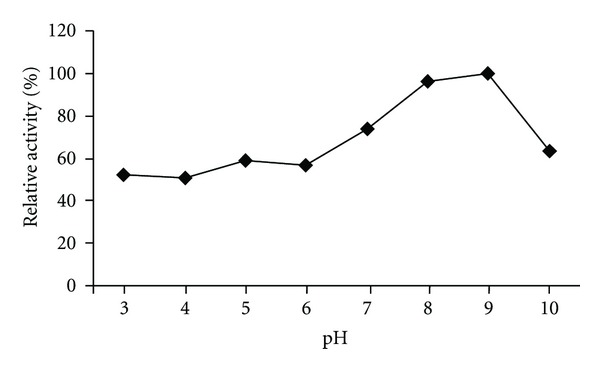
Effect of pH on chitinase activity.

**Figure 6:**
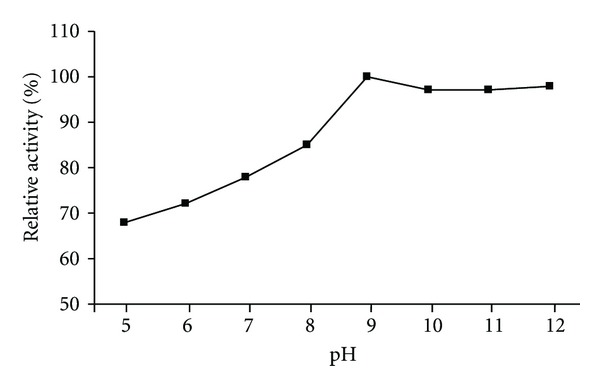
pH stability of chitinase.

The effects of various chemicals on enzyme activity were tested (Table 2) in the presence (at 1–5 mM concentrations) of Co^2+^, Zn^2+^, Ni^2+^, K^+^, Cu^2+^ enzyme activity was slightly enhanced by 16%, 18%, 32%, 44%, 56%, respectively. Wen et al. [3] reported that *Bacillus* sp. NCTU2 chitinase activity was enhanced by *≈*100% in the presence of 10 mM Ca^2+^. In the presence of Hg^2+^ and Cu^2+^ (at 10 mM) enzyme activity lost by 95%. Yuli et al. [27] reported that effect of metal ions on chitinase activity was quite different. In the presence of EDTA, SDS, HgCl_2_, ethyl=acetimidate the enzyme activity was partially inhibited by 7%, 7%, 11%, 20%, respectively, but (Table 2) other chemicals weakly affected enzyme activity. The enzyme molecular weight revealed as 125 and 50 kDa (Figure 2). Similar results were indicated by the other chitinase researchers by SDS-PAGE analyses: *Aeromonas* sp. chitinases was different in the range of 89–120 kDa [28], *Bacillus* sp. chitinase around 25–80 kDa [29], *Streptomyces* strain 68 chitinase, 25, 35, 67 and 200 kDa [30], *Bacillus* sp. NCTU2 chitinase 36.5 kDa [3], *Bacillus cereus* strain CH2 chitinase 15 kDa [25], and *Thermomyces lanuginosus* SY2 chitinase molecular size was estimated to be 48 kDa [26].

Chitinases have a broad spectrum of different industrial applications such as bio-control agents against plant pathogens (fungi) and insects (bio-insecticide friend with environment) and bioconversion of chitin waste to single-cell protein, Et-OH and fertilizer [14].

The thermostable chitinase had major advantages over industrial catalysis for its high activity at high temperature [26]. The properties of thermostable chitinase suggest that it could be useful for the chitin industry and biotechnological applications.

## Figures and Tables

**Table 1:** Morphological and physiological characteristics of chitinase-producing bacteria strain HBK-51.

Morphological characteristics	
Form	Rod
Gram stain	Positive
Spore	Terminal spore forming
Capsule	Negative
Motility	Positive
Physiological characteristics	
Catalase	Positive
Hydrolysis of starch	Positive
*β*-haemolysis	Positive
Cimons' Citrate	Positive
Hydrolysis of gelatin	Negative
Hydrolysis of l lecithin	Negative
Indol formation	Negative
VP-Test	Negative
Urease	Negative

**Table 2:** Influence of chemicals on chitinase activity.

Chemical	Con.	Act. (%)
control		100
DMSO	1%	92
	5%	92
EDTA	1 mM	95
	5 mM	93
Ethyl acetimidate	1 mM	90
	5 mM	80
SDS	1 mM	97
	5 mM	93
Phenol Gliaksol	1 mM	92
	5 mM	104
1,10-Phenanthroline	1 mM	90
	5 mM	100
N-Ethylmaleimide	1 mM	106
	5 mM	89
PMSF	1 mM	108
	5 mM	81
Urea	1 mM	106
	5 mM	80
Na-Sulphite	1 mM	98
	5 mM	96
MgCl_2_	1 mM	100
	5 mM	90
NaCl_2_	1 mM	97
	5 mM	93
BaCl_2_	1 mM	101
	5 mM	87
FeCl_3_	1 mM	81
	5 mM	106
MnCl_2_	1 mM	118
	5 mM	77
CuCl_2_	1 mM	127
	5 mM	155
CoCl_2_	1 mM	115
	5 mM	116
NiCl_2_	1 mM	132
	5 mM	123
ZnCl_2_	1 mM	118
	5 mM	106
KCl_2_	1 mM	127
	5 mM	144
CaCl_2_	1 mM	94
	5 mM	106
HgCl_2_	1 mM	93
	5 mM	89

Con: Concentration, Act: Activity.

The enzyme was incubated with various ions and reagents at room temperature for 30 min., then chitinase activity was assayed under standard conditions. The enzyme activity without any additive was taken as 100%.
